# OCTA‐Derived Retinal Biomarkers and Infarct Topography Improve Etiologic Classification of Recent Single Subcortical Infarction: A Nomogram Model

**DOI:** 10.1002/cns.70752

**Published:** 2026-01-19

**Authors:** Shuai Jiang, William Robert Kwapong, Yuying Yan, Tang Yang, Le Cao, Chen Ye, Junfeng Liu, Bo Wu

**Affiliations:** ^1^ Department of Neurology West China Hospital, Sichuan University Chengdu China; ^2^ Department of Neurology Xuanwu Hospital, Capital Medical University Beijing China

**Keywords:** branch atheromatous disease, cerebral small‐vessel disease, nomogram, recent single subcortical infarction, retinal microvasculature

## Abstract

**Background:**

Recent single subcortical infarction (RSSI) in lenticulostriate artery territories exhibits etiological heterogeneity. Misclassification risks persist due to overlapping neuroimaging features between cerebral small‐vessel disease‐related lacunar infarction (CSVD‐related LI) and branch atheromatous disease (BAD). We developed a nomogram that integrates retinal optical coherence tomography angiography (OCTA) metrics with infarct topography to improve etiological classification.

**Methods:**

Patients with RSSI were prospectively enrolled between December 2021 and December 2023. LASSO regression identified predictors for a logistic regression–based nomogram. Performance was evaluated via concordance index (C‐index), calibration curves, and decision‐curve analysis.

**Results:**

A total of 127 RSSI patients (86 CSVD‐related LI, 41 BAD) were included. Three variables—superficial vascular complex density, number of lesion slices, and proximal lesion location—were retained in the final model. The nomogram achieved a C‐index of 0.84 (95% CI, 0.80–0.89) versus 0.68 for conventional imaging, with superior net benefit across clinical thresholds (AUC 0.84 vs. 0.68, *p* < 0.001).

**Conclusion:**

The novel nomogram combining OCTA‐derived retinal biomarkers with infarct topography improves differentiation of BAD from CSVD‐related LI in RSSI patients and may facilitate etiology‐driven clinical decision‐making. External validation is needed for clinical implementation.

## Graphical abstract

- A nomogram integrating reduced superficial vascular complex density on OCTA with proximal and multi‐slice infarct topography improves differentiation of branch atheromatous disease (BAD) from CSVD‐related lacunar infarction (CSVD‐related LI) in recent single subcortical infarction (RSSI).
- This retina‐enhanced model outperforms conventional imaging alone and may facilitate etiology–driven clinical decision‐making.

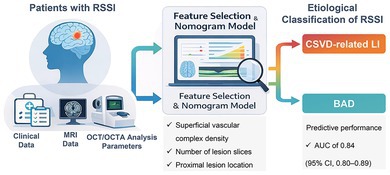

## 1 Introduction

Recent single subcortical infarctions (RSSI) with non‐stenotic parent arteries in the perforating territories demonstrate diverse underlying pathologies. Based on the seminal classification by Fisher and Caplan, RSSI etiology primarily bifurcates into two distinct entities: intrinsic cerebral small‐vessel disease‐related lacunar infarction (CSVD‐related LI) and branch atheromatous disease (BAD) resulting from parent artery pathology [1, 2]. BAD is believed to result from atherosclerotic plaques located at or near the origin of perforating arteries from the parent artery [3, 4]. These lesions may extend into the perforator lumen and lead to occlusion of the proximal penetrating branch. Consequently, BAD often produces larger and more longitudinally extensive infarcts, frequently involving multiple axial slices and located proximally within the perforator territory. Clinically, BAD has been associated with early neurological deterioration and a higher risk of unfavorable outcomes. In contrast, CSVD‐related LI typically results from intrinsic small‐vessel pathology such as lipohyalinosis, endothelial dysfunction, and blood–brain barrier impairment affecting distal perforating arterioles [5]. These infarcts are generally smaller, more distal, and commonly accompany other CSVD markers such as white‐matter hyperintensities, lacunes, or microbleeds. However, the lack of histopathological specimens and limited in vivo imaging capabilities hinders a precise understanding of the heterogeneous pathogenic mechanisms of RSSI.

To date, there has been little consistency in defining the size, location, and shape of the two distinct types of RSSI [3, 6]. To overcome these limitations, we implemented advanced high‐resolution magnetic resonance vessel wall imaging (VWI) to enable in vivo spatial mapping of pathological interactions between middle cerebral artery (MCA) atherosclerotic plaques and lenticulostriate artery (LSA) ostia [7]. This technological advancement facilitated the development of a novel VWI‐driven classification system that stratifies RSSI into BAD and CSVD‐related LI subtypes [5], aligning with original pathological descriptions [8]. Despite improved diagnostic precision, current VWI‐based differentiation protocols remain technically demanding, time‐consuming, and cost‐prohibitive, thus limiting their routine clinical application.

The retina, often described as an accessible “window” to the brain, may offer unique, noninvasive, and quantitative biomarkers for investigating the underlying etiology of cerebrovascular diseases [9, 10]. Biologically, the anatomy, physiology, and embryology of the neurons and microvasculature in the retina closely mirror those in the brain. Prior studies using traditional fundus photographs have demonstrated that focal arteriolar narrowing, venular dilation, and decreased fractal dimension were associated with lacunar stroke compared with other ischemic stroke subtypes [11, 12, 13, 14]. Emerging multimodal retinal imaging technologies, particularly optical coherence tomography (OCT) and OCT angiography (OCTA), now enable three‐dimensional quantification of microvascular architecture and hemodynamic profiling at capillary‐level resolution [15]. In contrast to the specialized, resource‐intensive MRI protocols needed for VWI, OCT and OCTA provides a quick, non‐invasive, and widely accessible optical imaging alternative, making it a practical tool for obtaining quantitative microvascular biomarkers in stroke patients.

An increasing body of evidence shows that quantitative changes in retinal structure (e.g., thinner retinal neuronal layers) and vasculature (e.g., lower microvascular densities, sparser microvasculature, and increased venular tortuosity) are associated with subclinical CSVD markers [16, 17], ischemic stroke, and related neurological and cognitive impairments [18, 19, 20, 21, 22, 23]. Our prior swept‐source OCTA investigation further revealed differential retinal microvascular patterns across ischemic stroke subtypes [24].

Accurate etiological stratification of RSSI subtypes holds therapeutic implications for targeted secondary prevention. Although prognostic nomograms integrating multimodal risk predictors are well established in cerebrovascular research [25, 26], no integrated model currently combines retinal imaging biomarkers, clinical profiles, and neuroimaging features to distinguish BAD from CSVD‐LI. To address this diagnostic challenge, we developed and validated a retina‐enhanced nomogram that integrates OCT and OCTA‐derived metrics with clinico‐radiological parameters for precise differentiation of these RSSI subtypes.

## 2 Methods

### 2.1 Participants

We identified participants from the prospective observational studies of RSSI at the Department of Neurology, West China Hospital of Sichuan University, between December 2020 and December 2023 [5, 7]. Briefly, the study prospectively recruited the first‐ever RSSI in the penetrating territory confirmed by diffusion‐weighted imaging (DWI). Evidence of cardioembolism, large‐artery disease, or non‐atherosclerotic vasculopathy was excluded. For the present study, we recruited all patients with the following inclusion criteria: [1] underwent VWI and OCT/OCTA imaging within 30 days of symptom onset; [2] no relevant MCA or basilar artery (BA) stenosis validated by magnetic resonance angiography (MRA) or computed tomography angiography (CTA).

Standardized cerebrovascular evaluation included 24‐h Holter monitoring, transthoracic echocardiography, and neurological assessment using the National Institutes of Health Stroke Scale (NIHSS) score. Demographic characteristics (age, sex) and vascular risk profiles (hypertension, diabetes, hyperlipidemia, smoking) were systematically documented.

### 2.2 MRI Imaging Protocol

Detailed brain imaging scanning protocols have been previously described [5]. In brief, all participants underwent a brain MRI at 3.0‐T MRI (MAGNETOM Trio, Siemens, Erlangen, Germany) equipped with a 32‐channel head coil. The imaging protocol included conventional T1‐weighted, T2‐weighted, fluid‐attenuated inversion recovery (FLAIR) images, DWI, three‐dimensional time‐of‐flight MRA (3D TOF‐MRA), and VWI sequences.

### 2.3 Image Analysis

MR images were evaluated using commercial software (OsiriX MD, Pixmeo SARL, Bernex, Switzerland) by two experienced neurologists (SJ and YY), blinded to all clinical information. VWI data were analyzed before reviewing conventional MR images. Discrepancies were resolved by consensus with a senior reviewer (BW) who was not involved in the initial readings. Details of interrater agreement for VWI imaging parameters were previously outlined [7].

### 2.4 Classification of BAD and CSVD‐Related LI by VWI

**FIGURE 1:**
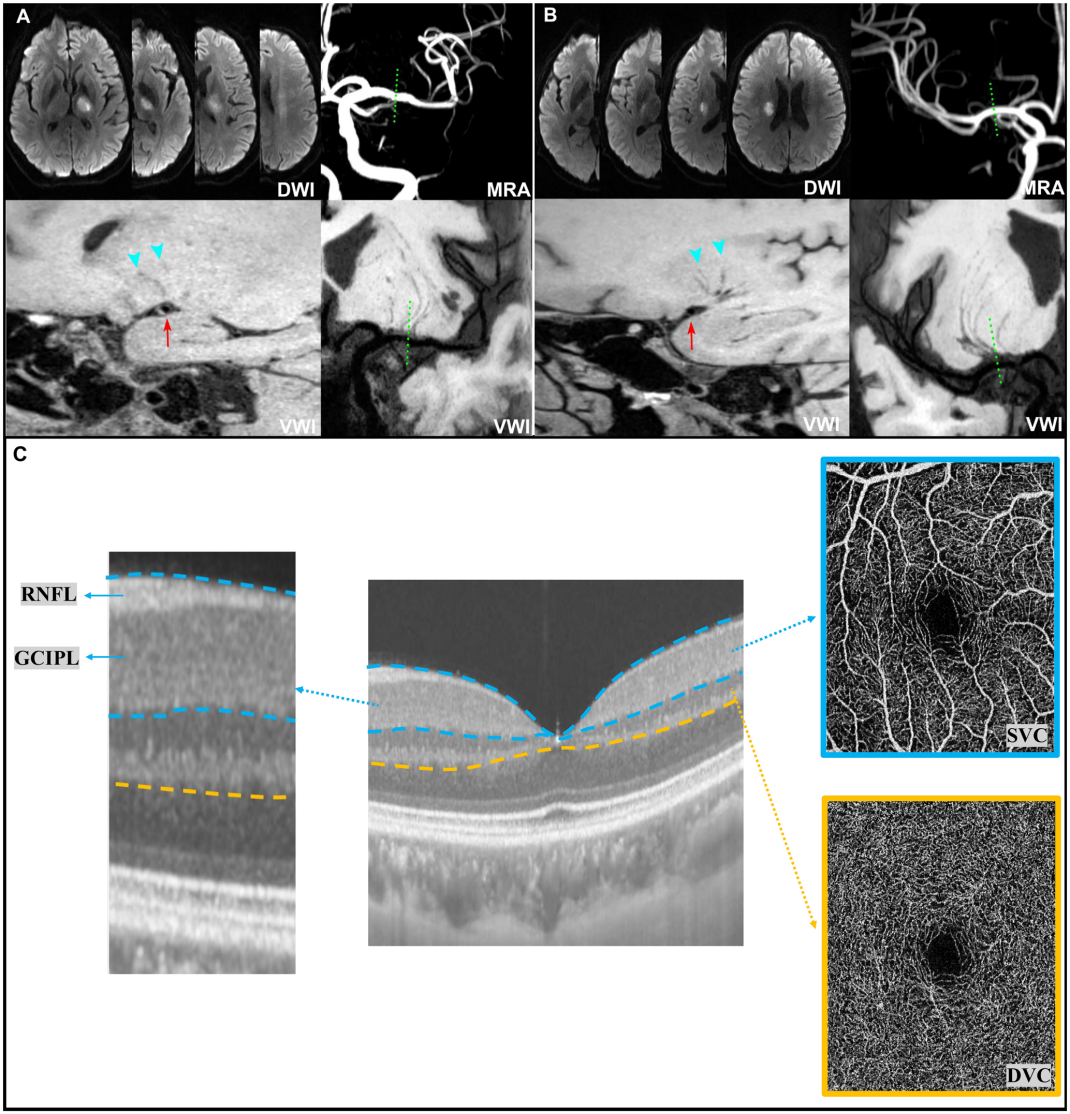
Classification of the etiological subtypes of RSSI based on high‐resolution magnetic resonance vessel wall imaging (VWI), and retinal microvascular and structural segmentation. (A) Branch atheromatous disease (BAD) DWI showed proximal RSSI involvement in 3 consecutive axial slices. Magnetic resonance angiography (MRA) showed no stenosis on the relevant middle cerebral artery (MCA). Curved multi‐planar reconstruction (curved‐MPR) showed a culprit plaque (red arrow) adjacent to the corresponding lenticulostriate arteries (LSAs) origins (dashed lines), demonstrated by the magnified image on cross‐section view with pruning LSAs (arrowheads). (B) Intrinsic cerebral small‐vessel disease‐related lacunar infarction (CSVD‐related LI) DWI showed distal RSSI involvement in 2 consecutive axial slices. Curved MPR showed no culprit plaque (red arrow) on the relevant MCA (dashed lines), demonstrated by the magnified image on cross‐section view with traceable LSAs (arrowheads). (C) Segmentation and representative images of retinal angiograms (retinal vascular plexuses) and retinal structural thicknesses. DVC, deep vascular complex; GCIPL, ganglion cell‐inner plexiform layer; RNFL, retinal nerve fiber layer; SVC, superficial vascular complex.

The etiologic classification of RSSI by VWI was assessed according to our previous method [5]. For RSSI in the MCA territory, multi‐planar reconstruction (MPR) and coronal minimum intensity projection (MinIP) were generated from VWI to simultaneously visualize MCA plaques and LSA origins. This approach enabled classification of infarcts as BAD (if plaques were located at or near the LSA origin) (Figure 1A) or CSVD‐related LI (if no plaque was seen in that region) (Figure 1B). For BA territory infarcts, BAD was defined as a culprit plaque located on the same or adjacent slice as the infarct, while CSVD‐related LI was diagnosed if no plaque or a remote plaque was present.

Lesion location was determined on DWI and classified as proximal or distal. A proximal lesion was defined as an infarct involving the basal surface supplied by the MCA or the ventral pontine surface supplied by the BA (Figure 1A). A distal lesion was defined as an infarct located in a distal region that did not extend to the basal or ventral pontine surface (Figure 1B) [5, 27]. CSVD MRI markers, including lacunes, cerebral microbleeds (CMBs), white‐matter hyperintensity (WMH), and perivascular spaces (PVS), were rated according to the STandards for ReportIng Vascular changes on nEuroimaging (STRIVE) consensus criteria [28]. An ordinal score ranging from 0 to 4 was established to reflect the summary CSVD score, as previously described [29].

### 2.5 Retinal Imaging

The retinal imaging examination used swept‐source optical coherence tomography (SS‐OCT) and OCT angiography (OCTA) (SS‐OCTA; SVision Imaging, Henan, China, version 2.1.016) as previously reported [17]. Retinal imaging was done in both eyes (left and right eyes) without pupil dilation. Structural OCT of the macular region was performed on a 3 × 3–mm area around the fovea. Macular retinal nerve fiber layer (RNFL) and ganglion cell–inner plexiform layer (GCIPL) thicknesses were measured automatically by the OCT tool (Figure 1C). Mean thicknesses (μm) of the RNFL and GCIPL were used in the analyses.

OCTA images were obtained with a raster‐scan protocol of 384 B‐scans that covered a 3 × 3–mm area around the fovea. En face angiograms of the superficial vascular complex (SVC) and deep vascular complex (DVC) were automatically generated (Figure 1C). The SVC and DVC were located in the inner two‐thirds and outer one‐third border of the GCIPL. Mean microvascular density (%) was used to measure the retinal microvasculature.

Images with retinal disorders such as age‐related macular degeneration, severe cataracts, and severe glaucoma were excluded. OCT and OCTA images with a signal quality of less than 7 were excluded. OCT and OCTA images met the OSCAR‐IB quality standards [30] and APOSTEL recommendations [31]. All angiograms were assessed by a trained, certified grader masked to participant characteristics (W.R.K). OCT and OCTA images with motion artifacts, such as irregular microvascular patterns or blurred segmentation, were excluded from further analysis. Participants with retinopathy, such as age‐related macular degeneration, severe cataracts, glaucoma, optic neuritis, and hemorrhages, were excluded. If a participant presented with any of these disorders in one eye, the other eye was used; if both eyes had the aforementioned disorders, the participant was excluded from the study.

### 2.6 Statistical Analyses

All statistical analyses were performed with R version 4.1.3. A two‐sided *p* < 0.05 was considered statistically significant. Data distribution was evaluated by visual inspection and the Kolmogorov–Smirnov test. Consecutive variables with normal distribution were expressed as mean and standard deviation (SD), while skewed distribution was expressed as median and interquartile ranges (IQR). Categorical variables were presented as frequencies and percentages (%). A generalized estimating equation (GEE) was used to compare OCT and OCTA features between BAD and CSVD‐related LI. Because OCT and OCTA metrics were obtained from both eyes, and measurements from the same participant are not statistically independent, we applied generalized estimating equation (GEE) models with the eye as the unit of analysis and the patient ID specified as the clustering variable. An exchangeable working correlation structure was used to account for inter‐eye correlation, and robust standard errors were estimated to provide valid inference. All GEE models were adjusted for age, sex, hypertension, diabetes mellitus, smoking status, and hyperlipidemia.

The least absolute shrinkage and selection operator (LASSO) method was employed to select the most predictive clinical variables and OCTA parameters for classifying RSSI subtypes. Subsequently, a multivariable logistic regression model was used to identify statistically significant predictors, which were then used to construct a nomogram. These features were presented as odds ratio (ORs), 95% confidence interval (95% CIs), and *p*‐value.

The calibration was plotted to assess the nomogram's overall discriminatory ability. Meanwhile, the Hosmer–Lemeshow test was used to examine how well the percentage of the observed probability matched the percentage of predicted probability over deciles of predicted risk (A *p*‐value > 0.05 indicated that the model is well calibrated). Harrell's concordance index (C‐index) and 1000 bootstrap replicates were computed to assess the model's internal validity. Decision curve analysis (DCA) was used to estimate the clinical value of the nomogram and traditional imaging model with the net benefit for multiple threshold probabilities. The area under the curve (AUC) of the receiver operating characteristic (ROC) was further used to compare the discrimination ability of the nomogram and traditional imaging model. Model accuracy was summarized using sensitivity, specificity, predictive values, and likelihood ratios.

## 3 Results

### 3.1 Patient Characteristics

We included 127 RSSI patients in the final analysis. 86 were classified as CSVD‐related LI, while 41 had BAD according to the VWI‐based classification. Among them, 105 (82.7%) were male, with a median age of 56 years (IQR, 49–62 years). The median interval between symptom onset and VWI was 5 days (IQR, 4–10 days). Table 1 presents the demographic characteristics, clinical features, and MRI and retinal imaging parameters of the study population.

**TABLE 1:** Baseline characteristics of study participants.

Variables	RSSI patients (*n* = 127)
*RSSI subtypes, n (%)*	
BAD	86 (67.7)
CSVD‐related LI	41 (32.3)
*Demographics*	
Male, *n* (%)	105 (82.7)
Age, y, median (IQR)	56 (49–62)
*Risk factors, n (%)*	
Hypertension	64 (50.4)
Diabetes	37 (29.1)
Hyperlipidemia	29 (22.8)
Current smoking	68 (53.5)
*Clinical data, median (IQR)*	
NIHSS on admission	3 (1–4)
Onset to baseline MRI time, days	5 (4–10)
Systolic blood pressure (SBP), mmHg	145 (131–161)
Diastolic blood pressure (DBP), mmHg	89 (78–96)
*RSSI location, n (%)*	
Basal ganglia/internal capsule	46 (36.2)
Corona radiata	60 (47.2)
Thalamus	5 (4.0)
Brainstem	16 (12.6)
*Infarct dimensions*	
DWI lesion axial diameter, mm, median (IQR)	15.6 (11.6–21.1)
Axial lesion diameter ≥ 1.5 cm, *n* (%)	67 (52.8)
DWI lesion volume, cm^3^	1.07 (0.48–2.91)
Number of lesion slices, median (IQR)	3 (2–4)
Number of lesion slices ≥ 3, *n* (%)	80 (63.0)
Proximal lesion, *n* (%)	65 (51.2)
*CSVD markers*	
PWMH, median (IQR)	1 (1–2)
PWMH (Fazekas 3), *n* (%)	22 (17.3)
DWMH, median (IQR)	1 (1–1)
DWMH (Fazekas 2–3), *n* (%)	17 (13.4)
Total WMH, median (IQR)	2 (2–3)
BG‐PVS, median (IQR)	2 (1–2)
CSO‐PVS, median (IQR)	2 (2–3)
Lacunes (≥ l), *n* (%)	49 (38.6)
Cerebral microbleeds (≥ l), *n* (%)	53 (41.7)
Summary CSVD score, median (IQR)	2 (1–3)
*OCT/OCTA derived measures*	
SVC, %	40.09 (35.64–43.25)
DVC, %	49.28 (47.06–51.69)
GCIPL, μm, median (IQR)	20.21 (19.11–21.52)
RNFL, μm, median (IQR)	78.97 (72.86–82.46)

*Note:* Data are presented as median (IQR) or number (%).

Abbreviations: BAD, branch atheromatous disease; BG, basal ganglia; BG‐PVS, basal ganglia perivascular spaces; CSO, centrum semiovale; CSVD, cerebral small‐vessel disease; DVC, deep vascular complex; DWI, diffusion‐weighted imaging; DWMH, deep white matter hyperintensity; GCIPL, ganglion cell‐inner plexiform layer; IQR, interquartile range; LI, lacunar infarction; NIHSS, National Institutes of Health Stroke Scale; PVS, perivascular spaces; PWMH, periventricular white matter hyperintensity; RNFL, retinal nerve fiber layer; RSSI, recent single subcortical infarction; SVC, superficial vascular complex; WMH, white matter hyperintensities.

**FIGURE 2:**
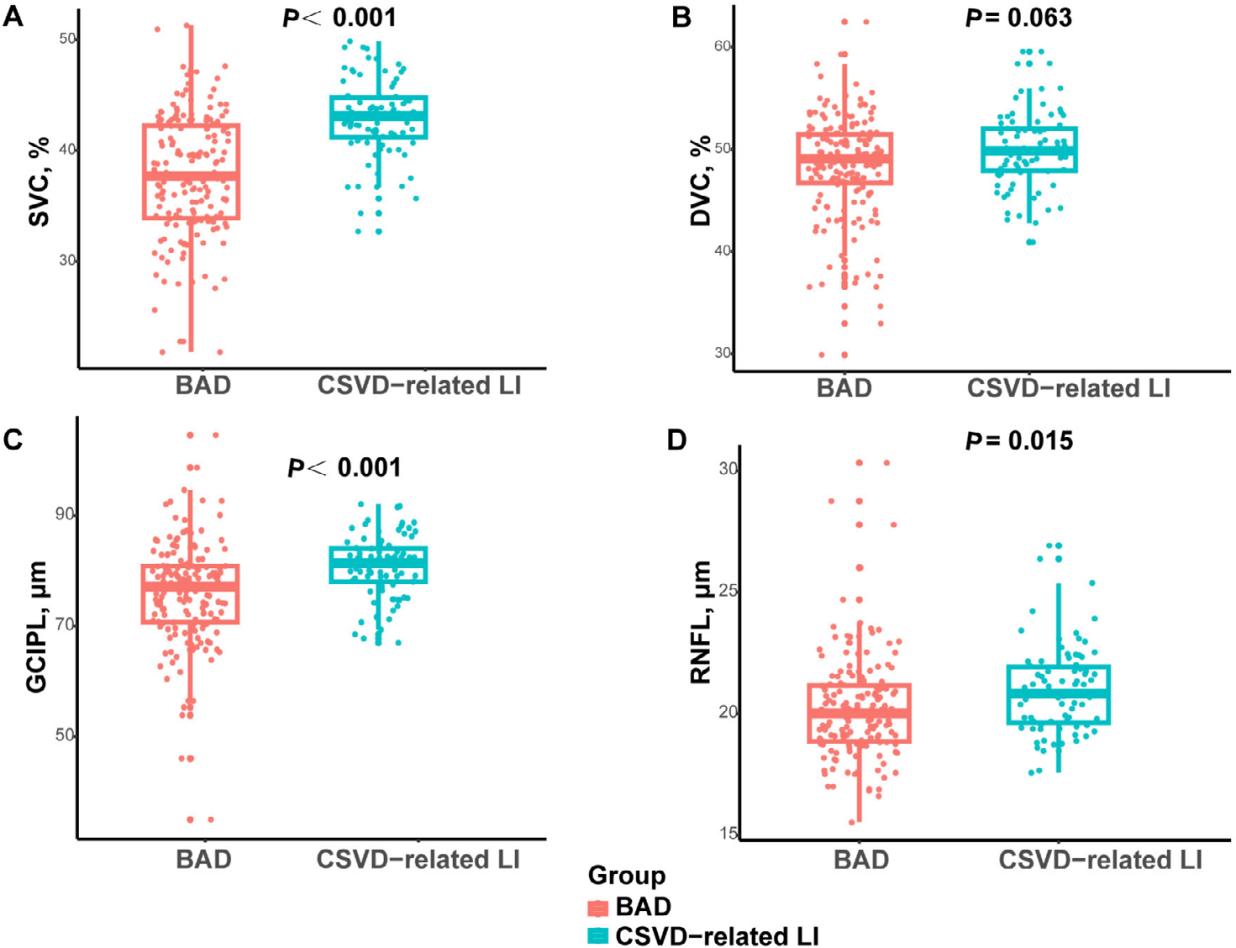
Comparison of OCT/OCTA metrics between BAD and CSVD‐related LI patients. SVC, superficial vascular complex; DVC, deep vascular complex; GCIPL, ganglion cell‐inner plexiform layer; OCT, optical coherence tomography; OCTA, OCT angiography; RNFL, retinal nerve fiber layer.

Figure 2 shows the comparison of OCT and OCTA metrics between BAD and CSVD‐related LI patients. Compared to those with CSVD‐related LI, patients with BAD exhibited lower SVC density (*p* < 0.001), thinner GCIPL thickness (*p* < 0.001), and reduced RNFL thickness (*p* = 0.015) after adjusting for age, sex, hypertension, diabetes mellitus, smoking status, and hyperlipidemia. No significant intergroup difference was observed in DVC density (*p* = 0.063).

### 3.2 Development of a Nomogram

Based on LASSO logistic regression analysis (Figure S1), three variables with nonzero coefficients were selected: SVC density, number of lesion slices, and proximal lesion location. The multivariate logistic regression model subsequently included these three variables, along with RNFL and GCIPL thicknesses.

**FIGURE 3:**
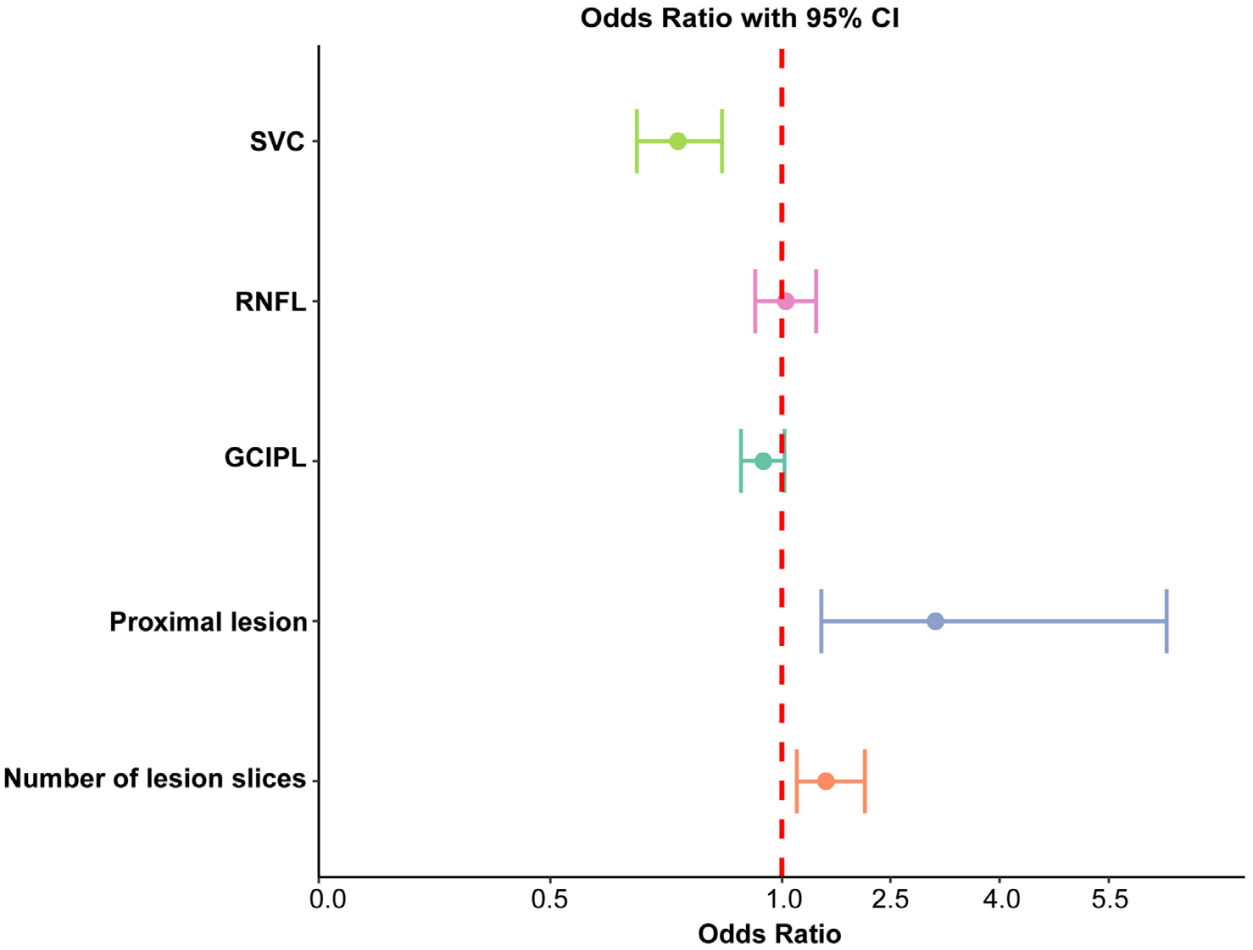
Forest plot of multivariate logistic regression analysis for differentiating BAD from CSVD‐related LI. BAD, branch atheromatous disease; CI, confidence interval; CSVD, cerebral small‐vessel disease; GCIPL, ganglion cell‐inner plexiform layer; LI, lacunar infarction; RNFL, retinal nerve fiber layer; SVC, superficial vascular complex.

**FIGURE 4:**
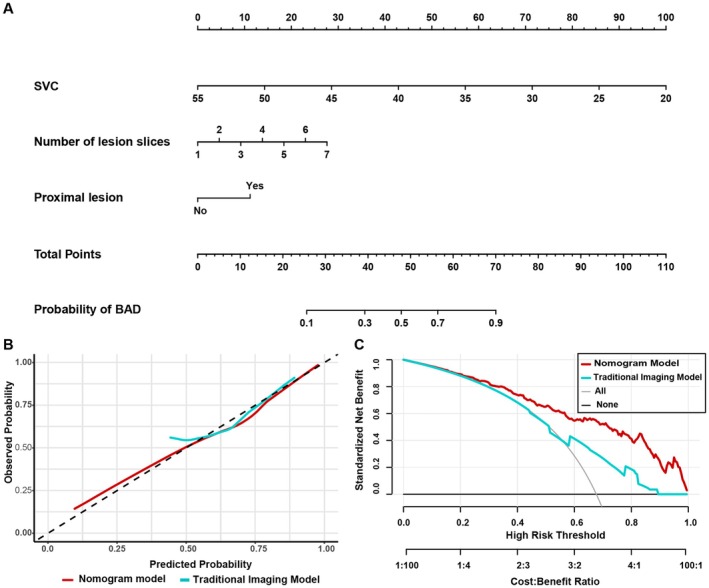
Nomogram for differentiating BAD from CSVD‐related LI and its predictive performance. (A) Nomogram predicting the probability of BAD in our RSSI cohort. (B) Calibration plot of actual probability versus predicted probability of BAD according to the nomogram and the traditional imaging models. (C) Decision curve analysis demonstrating the net benefit associated with the use of the nomogram model for predicting BAD in our RSSI cohort.

Multivariate logistic analysis found that SVC (OR, 0.751 [95% CI, 0.676–0.823]), proximal lesion (OR, 3.120 [95% CI, 1.555–6.429]), and number of lesion slices (OR, 1.607 [95% CI, 1.215–2.176]) could independently differentiate BAD from CSVD‐related LI (Table S1). Figure 3 presents a forest plot summarizing the regression results. These three independently associated variables were incorporated into a nomogram model for RSSI subtype classification (Figure 4A).

The nomogram demonstrated a C‐index of 0.86 and a bootstrap‐corrected C‐index of 0.84, indicating intense discrimination. In comparison, the traditional imaging model, which included only DWI‐derived topographic variables (number of slices and proximal lesion) without OCTA parameters, showed lower performance (C‐index of 0.78; bootstrap‐corrected C‐index of 0.68). The calibration curves for the probability of BAD subtype using this nomogram indicated good agreement between the prediction and observation in our cohort (Figure 4B), which was also confirmed by the Hosmer–Lemeshow test (*p* = 0.200). DCA revealed that the nomogram provided a higher net clinical benefit than the traditional imaging model across a wide range of threshold probabilities (Figure 4C), indicating superior clinical utility of the proposed model.

### 3.3 Nomogram‐Based Probability of Differentiating BAD From CSVD‐Related LI

**FIGURE 5:**
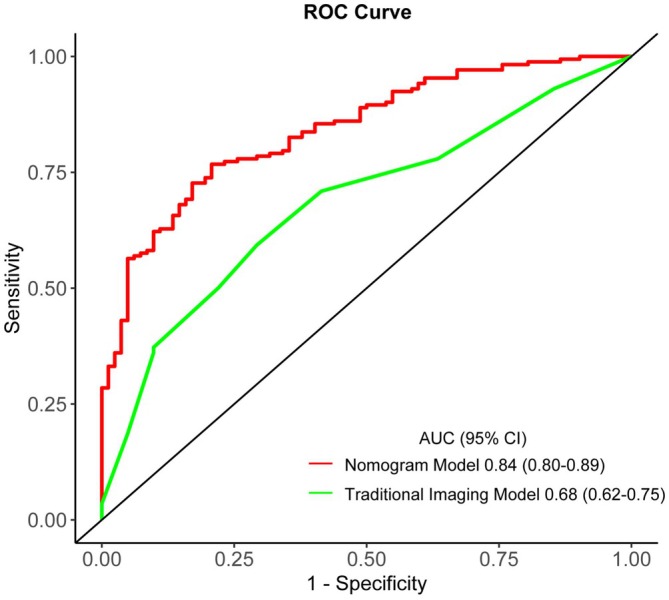
Receiver operating characteristic (ROC) curve of the nomogram and the traditional imaging models for differentiating BAD from CSVD‐related LI in our RSSI cohort.

Model performance was further validated using receiver operating characteristic (ROC) analysis. The nomogram model achieved an AUC of 0.84 (95% CI, 0.80–0.89), compared to an AUC of 0.68 (95% CI, 0.62–0.75) for the traditional imaging model (Figure 5), indicating the novel nomogram had higher predictive efficiency. The optimal cutoff value of the total nomogram scores was 57. At this threshold, the nomogram had a sensitivity of 89%, specificity of 62%, positive predictive value of 77%, negative predictive value of 79%, positive likelihood ratio of 2.33, and a negative likelihood ratio of 0.18 (Table S2).

## 4 Discussion

Differentiating the pathogenic mechanisms of the heterogeneous RSSI is clinically important. In this study, we developed and internally validated an individualized nomogram that combined two readily accessible clinical variables (number of lesion slices and proximal lesion) with one OCTA‐derived retinal microvascular parameter (SVC density) to distinguish BAD from CSVD‐LI in RSSI patients. Internal validation demonstrated robust discrimination (C‐index, 0.86) and calibration, exceeding conventional imaging models. Decision curve analysis confirmed clinical utility across a range of risk thresholds, supporting its implementation for personalized therapeutic strategies.

The retinocerebral homology positions retinal imaging as a reservoir of biomarkers for cerebrovascular diseases. While previous OCT/OCTA studies documented retinal thinning and microvascular rarefaction in stroke patients compared with age‐ and sex‐matched controls [18, 32, 33], our study extends these findings to RSSI subtyping. We showed that BAD had lower SVC density, thinner GCIPL, and thinner RNFL compared to CSVD‐related LI. However, in multivariate regression analysis, only SVC density remained an independent predictor for distinguishing BAD from CSVD‐related LI, indicating that reduced retinal microvascular density in the SVC may serve as a sensitive biomarker of early cerebral microcirculatory dysfunction in RSSI. The SVC, which encompasses retinal arterioles and venules supplying metabolically active ganglion cells [34], serves as a sensitive biomarker of tissue hypoxia and perfusion deficits. This aligns with reports linking SVC abnormalities to cerebral hypoperfusion and early neurological deterioration in ischemic stroke [18, 35], reinforcing its pathophysiological relevance. While reduced SVC density remained independently associated with BAD after multivariable adjustment, these findings should be interpreted as associative rather than causal. OCTA provides a cross‐sectional and indirect assessment of the retinal microvasculature, and SVC alterations are likely influenced by a range of systemic vascular and ocular factors, including global microvascular burden, ocular perfusion pressure, and medication effects. Therefore, reduced SVC density should not be considered specific to BAD; rather, it may represent a non‐invasive surrogate of generalized microvascular dysfunction or perfusion compromise that appears more pronounced in BAD compared with CSVD‐related LI. Longitudinal validation and mechanistic studies are warranted to further elucidate the temporal and causal relationships between retinal microvascular alterations and RSSI subtypes.

Neuroimaging characteristics provide critical insights into RSSI pathogenesis. BAD is pathognomonically characterized by atheromatous plaques at parent artery branch points, obstructing proximal perforators and causing larger infarcts [7]. In contrast, CSVD‐LI typically arises from distal lipohyalinosis, manifesting as small infarcts, with pathogenesis more closely linked to endothelial dysfunction and blood–brain barrier leakage than to direct arteriolar occlusion [36]. Supporting this hypothesis, our previous study has demonstrated that compromised cerebral perfusion is more frequent among patients with BAD and is more prone to early neurological deterioration compared to those with CSVD‐related LI [37]. Given the retina–brain vascular analogy, reduced SVC density in BAD patients may reflect similar microvascular pathologies leading to cerebral ischemia, and thus help differentiate it from CSVD‐related LI.

We also found that an increased number of axial lesion slices and proximal lesions are independently associated with BAD. However, the dimensional cutoff used to classify RSSI subtypes remains inconsistent across studies. Many studies define CSVD‐related LI as lesions < 15 mm in diameter and spanning < 3 axial slices, while BAD is characterized by lesions ≥ 15 mm in a single slice or involvement of ≥ 3 slices in the MCA territory [38, 39]. Compared with the axial lesion diameter, we suggested that the number of axial lesion slices provides a better appreciation of the discrepancy of infarct in predicting the pathogenic mechanism of RSSI, which was congruent with previous studies [40, 41]. Additionally, proximal lesions may result from atherosclerotic narrowing at the orifice or proximal segment of penetrating arteries and are a hallmark of BAD. In contrast, distal lesions are more often associated with lipohyalinosis and other CSVD features [42, 43]. Our findings support the clinical utility of infarct location in RSSI subtype classification.

Notably, no conventional CSVD markers were included in the final nomogram based on the VWI‐derived etiological framework. Traditional classification approaches posit that intrinsic CSVD‐related RSSI likelihood increases with concomitant CSVD burden (e.g., WMH, lacunes) [43, 44]. However, our findings align with prior VWI studies demonstrating comparable CSVD marker prevalence between plaque‐positive and plaque‐negative RSSI patients [42]. This discrepancy might be due to confounding vascular risk factors (e.g., older age, hypertension) that are more common in BAD patients than in those with CSVD‐related LI. Further studies with larger datasets are needed to validate this observation.

Conventional differentiation of BAD and CSVD‐related LI relies heavily on high‐resolution vessel wall imaging. Although this remains the reference standard, VWI requires 3‐Tesla MRI systems, specialized acquisition protocols, expert post‐processing, and substantial interpretation time. As such, its availability is limited, examination costs are high, and workflow integration into routine clinical practice is challenging. In contrast, OCT and OCTA are rapid, noninvasive, clinic‐based imaging modality that does not require contrast administration, radiation exposure, or advanced MR technology. Acquisition typically requires less than a minute per eye, and automated quantification enables reproducible and objective measurement of the retinal microvasculature in different plexuses and choroid. This makes OCT and OCTA feasible for widespread deployment in both inpatient stroke services and outpatient cerebrovascular clinics. Furthermore, in our study, a single OCTA‐derived microvascular metric—SVC density—combined with two readily accessible infarct topography features achieved strong discriminatory performance (C‐index, 0.84), outperforming models based on conventional imaging alone (C‐index, 0.68). Because high‐resolution VWI is not routinely performed, our model uses only widely available data (standard MRI lesion topography and OCTA) to infer RSSI etiology. In practice, this nomogram could help classify RSSI subtypes in settings lacking VWI.

This study has several limitations. First, the relatively small sample size was partly due to our strict inclusion criteria, excluding patients with proximal embolic sources. Moreover, only patients who underwent both VWI and OCT and OCTA were included. Second, as a single‐center study, selection bias may limit generalizability. Although the nomogram demonstrated good discrimination and calibration following internal bootstrap validation, the absence of external validation remains a key limitation. The present model should be considered exploratory and hypothesis‐generating rather than clinically definitive. Future studies incorporating larger, multicenter cohorts with independent validation datasets will be essential to confirm the reproducibility, generalizability, and clinical applicability of the proposed nomogram. Third, we did not analyze stroke outcomes (e.g., NIHSS progression or functional recovery) in this cohort, so it remains unknown how the nomogram score relates to clinical course. Future prospective studies should evaluate whether the nomogram can predict outcomes or guide management. Fourth, CSVD parameters were limited by visual ratings, whereas newer semi‐automated imaging analysis techniques could offer more precise quantification in future studies.

In conclusion, our multimodal nomogram synergizes lesion topography and retinal microvascular signatures to achieve precise RSSI subtyping. Incorporating such predictive models could help clinicians tailor interventions based on individual risk profiles. Prospective multicenter validation will further establish its translational value in improving RSSI management.

## Author Contributions

**Shuai Jiang, William Robert Kwapong**, and **Bo Wu:** study conception and design. **Tang Yang, Le Cao, Chen Ye**, and **Yuying Yan:** acquisition of data and clinical analysis. **Shuai Jiang, William Robert Kwapong**, and **Yuying Yan:** data interpretation and image analysis. **Shuai Jiang** and **William Robert Kwapong:** initial drafting of manuscript. **Bo Wu and Junfeng Liu:** revision of manuscript for important intellectual content.

## Funding

This work was supported by the National Natural Science Foundation of China (82271328, 82371322, 82301661), Sichuan Science and Technology Program (No. 2024YFFK0314), Post Doctor Research Project, West China Hospital, Sichuan University (2023HXBH007, 2024HXBH041), and the Noncommunicable Chronic Diseases–National Science and Technology Major Project (2023ZD0504900, 2023ZD0504903).

## Ethics Statement

Ethical approval (No. 2020[922]) was obtained from the Institutional Review Board of West China Hospital, Sichuan University, with written informed consent from all participants.

## Conflicts of Interest

The authors declare no conflicts of interest.

## Supporting information


**Figure S1:** Features selection using the LASSO binary logistic regression model. (A) The LASSO coefficient profiles of the 35 features. A coefficient profile plot was produced against the log (lambda) sequence. (B) Parameters selection in the LASSO model used tenfold cross‐validation via the minimum criterion. Partial likelihood deviation (binomial deviation) curves and logarithmic (lambda) curves were plotted. Use the minimum standard and 1se (1‐SE standard) of the minimum standard to draw a vertical dashed line at the optimal value. The optimal lambda produced three nonzero coefficients.

**Table S1:** Multivariate logistic regression analyses differentiating BAD from CSVD‐related LI.
**Table S2:** Accuracy for the prediction score of the nomogram for differentiating BAD from CSVD‐related LI in our RSSI cohort.

## Data Availability

The data that support the findings of this study are available from the corresponding author upon reasonable request.
