# Supplementary figures and images for: OCTA‐Derived Retinal Biomarkers and Infarct Topography Improve Etiologic Classification of Recent Single Subcortical Infarction: A Nomogram Model
Source: CNS Neurosci Ther. 2026 Jan 19;32(1):e70752. doi: 10.1002/cns.70752 (PMC12813863; doi:10.1002/cns.70752)

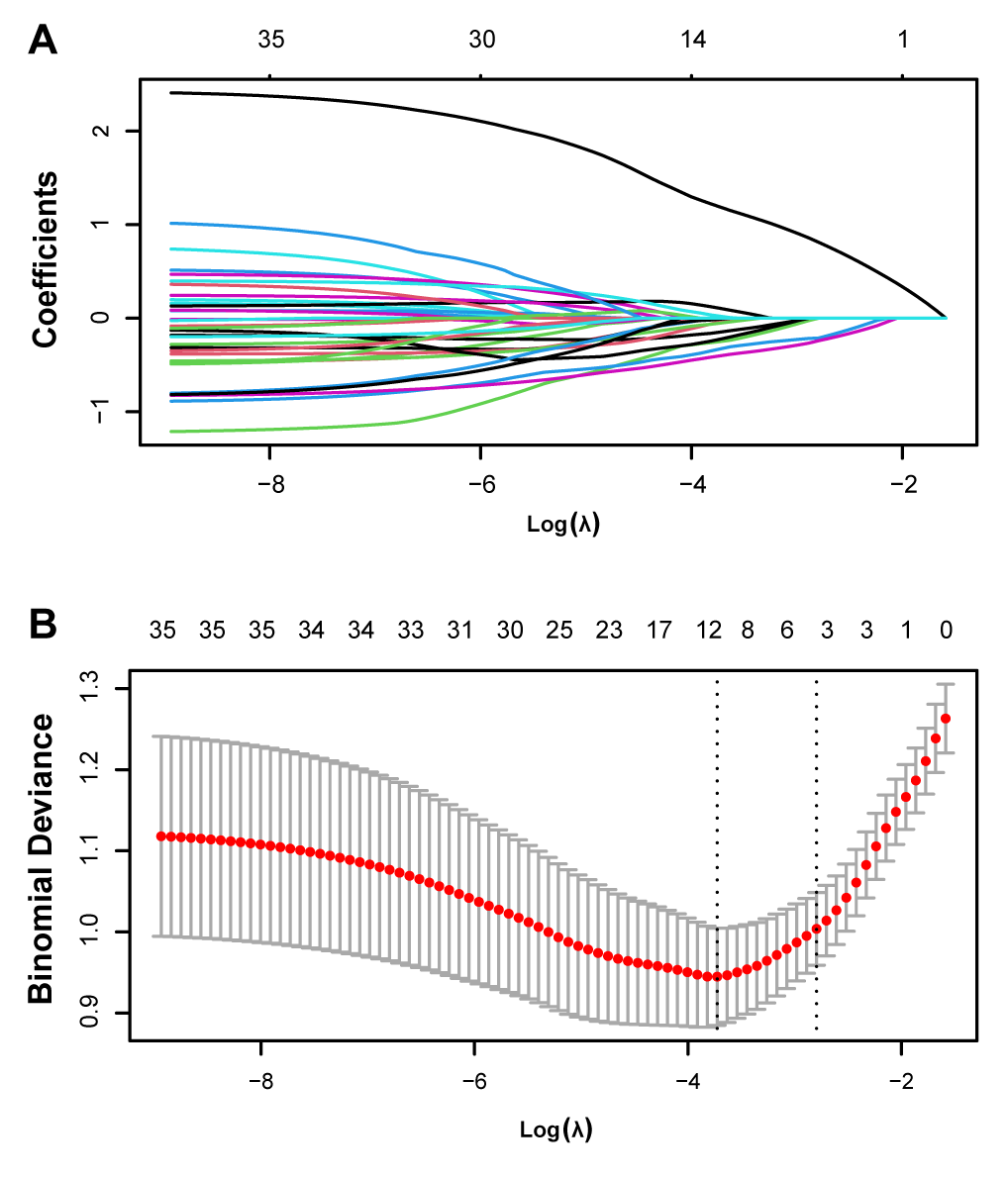

Supplement: Supplementary file 1 — Figure S1: Features selection using the LASSO binary logistic regression model. (A) The LASSO coefficient profiles of the 35 features. A coefficient profile plot was produced against the log (lambda) sequence. (B) Parameters selection in the LASSO model used tenfold cross‐validation via the minimum criterion. Partial likelihood deviation (binomial deviation) curves and logarithmic (lambda) curves were plotted. Use the minimum standard and 1se (1‐SE standard) of the minimum standard to draw a vertical dashed line at the optimal value. The optimal lambda produced three nonzero coefficients. [file CNS-32-e70752-s002.tif]
