# Supplementary material for: OCTA‐Derived Retinal Biomarkers and Infarct Topography Improve Etiologic Classification of Recent Single Subcortical Infarction: A Nomogram Model
Source: CNS Neurosci Ther. 2026 Jan 19;32(1):e70752. doi: 10.1002/cns.70752 (PMC12813863; doi:10.1002/cns.70752)
Supplement: Supplementary file 2 — Table S1: Multivariate logistic regression analyses differentiating BAD from CSVD‐related LI. Table S2: Accuracy for the prediction score of the nomogram for differentiating BAD from CSVD‐related LI in our RSSI cohort. [file CNS-32-e70752-s001.docx]

**OCTA-Derived Retinal Biomarkers and Infarct Topography Improve Etiologic Classification of Recent Single Subcortical Infarction: A Nomogram Model**

Shuai Jiang^1,^*, William Robert Kwapong ^2,^*, Yuying Yan^1^, Tang Yang^1^, Le Cao^1^, Chen Ye^1^, Junfeng Liu^1,#^ and Bo Wu^1,#^

1. Department of Neurology, West China Hospital, Sichuan University, Chengdu, China

2. Department of Neurology, Xuanwu Hospital, Capital Medical University, Beijing, China.

#Correspondence to

Bo Wu, Department of Neurology, West China Hospital, Sichuan University, No. 37, Guo Xue Xiang, Chengdu 610041, China. Email: dr.bowu@hotmail.com or Junfeng Liu, Department of Neurology, West China Hospital, Sichuan University, No. 37, Guo Xue Xiang, Chengdu 610041, China. Email: junfengliu225@outlook.com.

*These authors contributed equally to the manuscript.

**SUPPLEMENTAL MATERIALS**

**Table S1 Multivariate logistic regression analyses differentiating BAD from CSVD-related LI**

| **Variables** | **OR (95% CI)** | ***p* Value** |
| --- | --- | --- |
| SVC, % | 0.751(0.676~0.823) | ＜0.001 |
| RNFL, µm | 1.180(0.945~1.482) | 0.147 |
| GCIPL, µm | 0.967(0.901~1.035) | 0.344 |
| Proximal lesion | 3.120(1.555~6.429) | 0.002 |
| Number of lesion slices | 1.607(1.215~2.176) | 0.001 |

Abbreviations: CI = confidence interval; OR = odds ratio; SVC: superficial vascular complex; GCIPL: ganglion cell-inner plexiform layer; RNFL: retinal nerve fiber layer; BAD: branch atheromatous disease; CSVD: cerebral small vessel disease; LI: lacunar infarction.

**Table S2. Accuracy for the prediction score of the nomogram for differentiating BAD from CSVD-related LI in our RSSI cohort.**

| **Variable** | **Value (95% CI)** |
| --- | --- |
| Area under the ROC curve, C index | 0.84 (0.80 - 0.89) |
| Cutoff score | 57 |
| Sensitivity, % | 0.89 (0.82 - 0.93) |
| Specificity, % | 0.62 (0.52 – 0.71) |
| Positive predictive value, % | 0.77 (0.70 – 0.83) |
| Negative predictive value, % | 0.79 (0.69 - 0.87) |
| Positive likelihood ratio | 2.33 (1.81 - 2.99) |
| Negative likelihood ratio | 0.18 (0.11 - 0.30) |

BAD: branch atheromatous disease; CSVD: cerebral small vessel disease; LI: lacunar infarction; ROC: receiver operating characteristic; CI: confidence interval.

**Figure S1. Features selection using the LASSO binary logistic regression model.** (A) The LASSO coefficient profiles of the 35 features. A coefficient profile plot was produced against the log (lambda) sequence. (B) Parameters selection in the LASSO model used tenfold cross-validation via the minimum criterion. Partial likelihood deviation (binomial deviation) curves and logarithmic (lambda) curves were plotted. Use the minimum standard and 1se (1-SE standard) of the minimum standard to draw a vertical dashed line at the optimal value. The optimal lambda produced three nonzero coefficients.

LASSO: least absolute shrinkage and selection operator; SE: standard error.
